# Aging, mental illness and COVID-19: Focusing research on vulnerable populations

**DOI:** 10.1016/j.nbas.2021.100007

**Published:** 2021-01-22

**Authors:** Joshua Gordon, Jovier D. Evans

**Affiliations:** National Institute of Mental Health, National Institutes of Health, Bethesda, MD, USA

The world is aging. There are now more individuals over the age of 65 than children under the age of 5. The population of older individuals with serious mental illness (SMI) is expected to grow substantially over the next two decades. Given the high rate of disability associated with these illnesses, much work is needed to understand both risk and resilience factors across the lifespan. In addition, given the lack of geriatric mental health care experts and clinicians and the high public health burden, more research is needed to maximize health span, the portion of life spent in good health, not just life span. The COVID-19 pandemic poses additional challenges to older individuals, especially those with SMI and from minority and vulnerable communities. These challenges must be met by research that pays specific attention to aging individuals with SMI, aimed at expanding access to evidence-based solutions to those most in need.

## 1 Aging in the context of SMI

Evidence has mounted that people with SMI are at increased risk of medical illnesses and premature mortality from natural causes, with decreased life expectancy of up to 25 years [1] from the general population. Most of these medical illnesses are those that are seen with advanced age, such as cardiovascular disease and diabetes. This has raised the notion that SMIs are associated with accelerated biological aging that is seen in both the brain and throughout the body. Research and clinical efforts have been focused on ameliorating comorbid somatic concerns, as well as understanding the biological processes that may moderate or exacerbate these changes in midlife to prevent negative consequences later in life [2].

The geoscience hypothesis posits that since aging physiology plays a role in many – if not all – chronic diseases, addressing aging physiology as a treatment target, will allow a reduction or delay in the appearance of multiple chronic diseases. Older adults are often afflicted by multiple comorbidities and while recent progress in addressing individual diseases has led to an increase in life expectancy, this has not always been accompanied by a parallel increase in health span [3]. Given the importance of understanding these aging mechanisms, work focused on biological aspects of cellular senescence, inflammation, and oxidative stress may point to potential treatment targets.

In contrast, however, many SMIs are at least partially neurodevelopmental in nature, and the accelerated aging and morbidity would call for an integration of these approaches prior to late life. In addition, people with mood and anxiety disorders are also at higher risk for neurodegenerative disorders in late life. Promoting intervention and recovery among this population may have a benefit in preventing the development of dementia in later life. Neuropsychiatric symptoms in dementia also are poorly understood and treated and can lead to high costs on the public health system.

Another serious risk in late life is suicide. Suicide is the tenth leading cause of death in the United States. In late adulthood, suicide rates are higher than in any other age group and have risen over 40% in the past ten years [4]. There is an urgent need to understand the risk factors and mechanisms that contribute to this elevated risk and the need to develop and promote effective interventions for this vulnerable population. Possible avenues concern the study of cognitive neuroscience approaches to decision making in older adults, and the promotion of social integration and connectedness among older people, particularly older men.

An alternative research pathway in the study of older adults with mental illness is to determine protective and resilience factors in this group. The study of older adults with SMI that are thriving and or resilient to some of these more degenerative mechanisms may point to potential new targets for intervention. Studying individuals with SMI who have made it to old age may be a way to understand what sort of protective factors are at play to slow or halt the negative consequences of aging. A broader course of action in the study of brain aging concerns more work with centenarians with little or no disability. These oldest old may point to the examination of lifestyle and or genetic factors associated with both extended lifespans, but also extended brain span.

## 2 Mental health in the elderly during the pandemic and beyond

The COVID-19 pandemic has had an extremely detrimental impact on older, vulnerable adults. Issues with regard to health care access, the current state of elder care, and the significant risk to people with established comorbidities point to an urgent need to improve the broader health care system. Additionally, with the need to shelter in place, the pandemic has focused attention on issues of social isolation, social disconnection and loneliness, particularly among older people. Prior to the pandemic, older adults were more likely to be socially isolated than younger people. Indeed, loneliness was the focus of a National Academies report [5] on the significant negative health consequences associated with social disconnection and loneliness. Nearly 25% of Americans over 65 in community settings are socially isolated, and a significant number of adults over the age of 45 report feeling lonely. This social isolation has been associated with significant morbidity and mortality among the elderly. The report noted the need for more work to develop an appropriate evidence base for interventions targeting loneliness in order to translate research into effective clinical care, and more work in the community aimed at harnessing both healthcare resources and community-based networks to improve social connectedness. Accordingly, the NIMH seeks to support research that examines both neural and environmental mechanisms associated with social disconnection, social isolation, and loneliness. In addition, research that addresses new and potential targets for treatment and intervention are needed if we are to address this issue.

The disproportionate impacts of the COVID-19 pandemic on minority communities have highlighted another pre-pandemic problem facing older adults. The associated medical comorbidities and financial constraints that accompany older age disproportionately affect people of color, impacting resilience and access to care in the context of the pandemic. These disparities highlight the imperative to do more research in underserved and vulnerable populations to improve our understanding of the biological and environmental factors associated with poor health across the lifespan. Such research must be squarely focused on improving health and well-being in all of the communities we serve.

An additional complication of COVID-19 that requires further study is the long-term impact of COVID-19 among so called “long haulers”, people who have recovered initially, but are still suffering lingering effects. The long terms effects of the COVID-19 virus will not only affect physical health but will undoubtedly also raise risk of comorbid psychiatric and behavioral consequences. Among people with existing mental health issues, the additional health risks may be particularly severe. All of these issues will have an impact on longitudinal studies of both brain and body health over the lifespan and will require further investigation.

Novel treatments and interventions that can be delivered via new and innovative methods have the potential to mitigate some of the impacts of COVID-19. Most treatments have become virtual over the past several months, utilizing both telehealth and computer approaches to behavioral interventions. Given the paucity of mental health care providers that specialize in treatment for older adults, these practices have the potential to extend the reach of evidence-based approaches, even after the conclusion of the pandemic. Maximizing this reach will require additional research that ensures the efficacy of these approaches specifically in older individuals, as well as implementation research aimed at understanding the efforts that will aid adoption, including training and equipping both providers and clients. Moreover, the shortage of mental health professionals (particularly those with geriatric expertise) also calls for the development of treatments and interventions that can be performed by other health care professionals and paraprofessionals. Such efforts must ensure competent, culturally appropriate services can be delivered to the vast majority of older adults who may not seek or have access to specialty mental health care.

In summary, there is an urgent need to both understand brain aging in general, but also how these changes in brain aging relate to mental illness risk and prevention, especially in the age of COVID. Among those with SMI, more work is needed to understand the intersection of both mental illness and the biology of aging. In addition, more research is needed to understand basic mechanisms of aging as they relate to psychopathology across the lifespan, and potential targets for prevention and treatment. The current COVID-19 pandemic also spotlights the need for more work with vulnerable populations and the need for widespread, innovative treatment outreach that will be of benefit to the largest group of people in need. The concomitant benefits to either prevention of negative mental health outcomes, or appropriate strategies to intervene to ameliorate these negative states among older people with SMI is a key component of the mission of the NIMH.

## References

[1] Viron M.J., Stern T.A. (2010). The impact of serious mental illness on health and healthcare. Psychosomatics.

[2] Bersani F.S., Mellon S.H., Reus V.I., Wolkowitz O.M. (2019). Accelerated aging in serious mental disorders. Curr Opin Psychiatry.

[3] Sierra F. (2019). Gerosicence and the challenges of aging societies. Aging Med.

[4] Hedegaard H., Curtin S.C., Warner M. (2018).

[5] National Academies of Sciences, Engineering, and Medicine (2020).

